# New insights on the optical properties and upconversion fluorescence of Er-doped CoAl_2_O_4_ nanocrystals

**DOI:** 10.1039/d3ra07928g

**Published:** 2024-01-24

**Authors:** N. T. Kien, V. D. Lam, P. V. Duong, N. T. Hien, N. T. Luyen, P. V. Do, N. T. Binh, N. X. Ca

**Affiliations:** a Institute of Science and Technology, TNU-University of Sciences Thai Nguyen Vietnam hiennt@tnus.edu.vn canx@tnus.edu.vn; b Graduate University of Science and Technology, Vietnam Academy of Science and Technology Hanoi Vietnam; c Institute of Physics, Vietnam Academy of Science and Technology Hanoi Vietnam tbnguyen@iop.vast.ac.vn; d Thuyloi University 175 Tay Son, Dong Da Hanoi Vietnam

## Abstract

In this study, Er-doped CoAl_2_O_4_ nanocrystals (NCs) were synthesized *via* co-precipitation. All the NCs were crystallized in the form of a single phase with a spinel structure and Er^3+^ ions replaced Al^3+^ ions in the formation of the CoAl_2−*x*_Er_*x*_O_4_ alloy structure. The optical characteristics of the Er^3+^ ion-doped CoAl_2_O_4_ NCs were thoroughly investigated by analyzing both the UV-VIS and photoluminescence spectra, using the Judd–Ofelt theory. The effect of Er doping content on the luminescent properties of the CoAl_2_O_4_ pigment (using lasers emitting at wavelengths of 413 and 978 nm) has been studied. The values of Judd–Oflet intensity parameters (*Ω*_2_, *Ω*_4_, and *Ω*_6_) were determined from the absorption spectra using the least square fitting method. The J–O parameters were calculated and compared with those of other host materials; the values of the *Ω*_2_, *Ω*_4_, and *Ω*_6_ parameters decreased with an increase in Er concentration. This suggests that the rigidity and local symmetry of the host materials become weaker as the concentration of Er^3+^ ions increases. The highest value of the *Ω*_2_ parameter, when compared with *Ω*_4_ and *Ω*_6_, suggests that the vibrational frequencies in the given samples are relatively low. The upconversion fluorescence phenomenon was observed and explained in detail under an excitation wavelength of 978 nm when the excitation power was varied.

## Abstract 2

Er-doped CoAl_2_O_4_ NCs were synthesized *via* co-precipitation; all were crystallized in the form of a single phase with a spinel structure and Er^3+^ ions replaced Al^3+^ ions in the formation of the CoAl_2−*x*_Er*_x_*O_4_ alloy structure.

## Graphical abstract

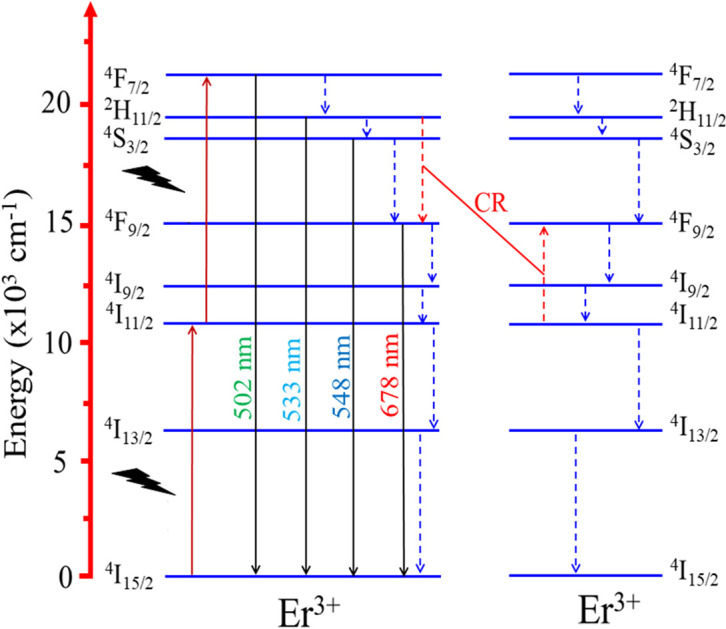

## Introduction

Spinel-structured oxides with the general chemical formula AB_2_O_4_ are widely employed as ceramic pigments, magnetic materials, catalysts, and more.^1,2^ Among all spinel oxides, cobalt aluminate (CoAl_2_O_4_), also known Thenard's blue, is an inorganic blue pigment. Within this pigment, Co^2+^ ions are situated in tetrahedral positions, whereas Al^3+^ ions occupy octahedral positions.^3^ This special CoAl_2_O_4_ blue pigment has played an important role in color printing technology for materials such as plastics, paints, glass, enamels, and ceramics. In addition to its brilliant blue color, this inorganic CoAl_2_O_4_ pigment exhibits remarkable resistance to both acids and alkalis, along with enhanced thermal stability.^4^

CoAl_2_O_4_ is a superparamagnetic single-domain crystal material at room temperature.^5^ Research has mainly focused on the absorption properties of CoAl_2_O_4_ and there are very few studies on the fluorescence properties of CoAl_2_O_4_ owing to its weak emission ability. The vibrancy of CoAl_2_O_4_ pigments can be compromised because of their susceptibility to contain Co_3_O_4_ phase impurities, which impart a black hue.^4,5^ The coloration of a ceramic pigment is intricately linked to factors such as its composition, crystal structure, grain size, and micro-morphology.^2–4^ The impact of the preparation process of the ceramic pigment on its microstructure and color performance is important for enhancing its chromatic richness and broadening its range of applications.^3–5^ Some studies have doped transition metals and rare earth ions into the CoAl_2_O_4_ host to change the color and enhance the luminescence of the material. Y. Tong *et al.* doped Eu^3+^ ions into a CoAl_2_O_4_ host and found that CoAl_1.95_Eu_0.05_O_4_ NCs can be considered a good “colored cool pigment” candidate for use in surface coating applications.^6^ Ce and Mn co-doped CoAl_2_O_4_ NCs were successfully synthesized using a novel wet chemical method.^7^ The introduction of Ce and Mn ions into CoAl_2_O_4_ enhanced its photoluminescence properties without altering the cubic structure of the host. The improved photoluminescence performance of Ce and Mn co-doped CoAl_2_O_4_ is not attributed to energy transfer between Ce^4+^ and Mn^4+^; rather, it is influenced by surface or impurity defects. Strontium-doped cobalt aluminate NCs were fabricated using Co_1−*x*_Sr_*x*_Al_2_O_4_l-alanine as a fuel in an ignition cycle.^8^ CoAl_2_O_4_ and Ni-doped CoAl_2_O_4_ were synthesized using the pechini route.^9^ The breakdown of the colored and colorless organic dyes under natural sunlight was investigated using the prepared photocatalysts. Ni-doped CoAl_2_O_4_ exhibited greater photocatalytic activity than undoped CoAl_2_O_4_.

Up-conversion luminescence is a phenomenon in which light is emitted at a wavelength shorter than the wavelength of the incident radiation. The up-conversion emission mechanism is often observed in certain materials, such as host materials doped with rare-earth ions (*e.g.*, erbium, ytterbium, and holmium). This phenomenon originates from the absorption of two or more photons and is commonly observed in rare-earth ions, that possess intricate energy-level structures.^10,11^ Rare earth elements exhibit fluorescence spectra at various energy levels, providing a wide range of wavelengths, making them valuable luminescent materials.^12^ Er^3+^ ions are particularly intriguing because they can achieve up-conversion luminescence without the need for a sensitizer like some other rare earth ions.^13,14^ The intricate configuration of their electronic energy levels enables the detection of transitions at various wavelengths. When stimulated by infrared radiation, Er^3+^ ions exhibit diverse color emissions, rendering them significant luminescent ions in the mid-infrared spectrum, and leading to a multitude of applications.^15^ Er^3+^ ions can emit both green and red light when excited by infrared radiation.^16,17^ Up-conversion materials have applications in various fields, including photovoltaics, bioimaging, and laser technology.

Some studies have been conducted on the optical properties of rare earth ions-doped CoAl_2_O_4_ NCs.^6–9^ However, there has been no research related to the optical parameters of CoAl_2_O_4_ NCs doped with Er^3+^ ions, particularly using the Judd–Ofelt theory and their upconversion fluorescence. In this study, for the first time the local environment around Er^3+^ ions, the bond between Er^3+^ and ligands, and the local symmetry of the host material CoAl_2_O_4_ were studied using J–O theory. The nature of the upconversion fluorescence mechanism of Er ion in the CoAl_2_O_4_ host (using an excitation wavelength of 978 nm) has been studied and quantitatively calculated by changing the excitation power. The effect of Er doping content on the luminescent properties of the CoAl_2_O_4_ pigment (using lasers emitting at wavelengths of 413 and 978 nm) has been studied. The development of these new ceramic pigments has scientific significance and practical value.

## Experimental description

### Synthesis of Er-doped CoAl_2_O_4_ spinel nanocrystals

**Fig. 1:**
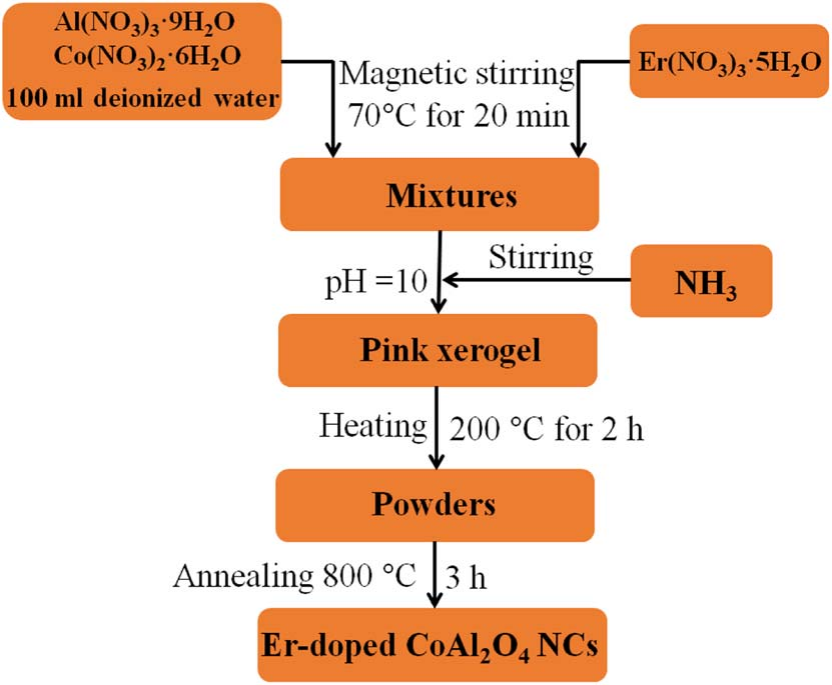
Schematic flow chart for the synthesis of Er-doped CoAl_2_O_4_ NCs.

In our experiment, 0.06 mol Al(NO_3_)_3_·9H_2_O and 0.03 mol Co(NO_3_)_2_·6H_2_O were dissolved in 100 ml deionized water under magnetic stirring at 70 °C for 20 min. Er(NO_3_)_3_·5H_2_O was then added to the above solution. The Er^3+^ concentration depended on the Er^3+^/Al^3+^ ratio. Then NH_3_ solution was added to the above solution to adjust the pH value to approximately 10 and continued stirring the solution at 70 °C until a pink xerogel formed. The xerogel was then heat treated at 200 °C for 2 h to remove a significant portion of the organic solvent and water. Finally, the dried xerogel was ground into a fine powder and calcined for 3 h at 800 °C in air. Schematic flow chart for the synthesis of Er-doped CoAl_2_O_4_ NCs is observed in Fig. 1. The evolution process can be depicted by the following reactions:

1 NH_3_ + H_2_O → NH_4_^+^ + OH^−^

2 Al^3+^ + 3OH^−^ → Al(OH)_3_

3 Al(OH)_3_ + OH^−^ → 2H_2_O + AlO_2_^−^

4 Co^2+^ + 2OH^−^ → Co(OH)_2_

5 Er^3+^ + 3OH^−^ → Er(OH)_3_

6 2Al(OH)_3_ → Al_2_O_3_ + 3H_2_O

7 Co(OH)_2_ → CoO + H_2_O

8 2Er(OH)_3_ → Er_2_O_3_ + 3H_2_O

9 2CoO + (2−*x*)Al_2_O_3_ + *x*Er_2_O_3_ → 2CoAl_2−*x*_Er_*x*_O_4_

### Characterization

The crystal structures of the synthesized nanocrystals (NCs) were analyzed using an X-ray diffraction (Siemen, D5005) equipped with a Cu-Kα radiation source (*λ* = 1.5406 Å) within the 2*θ* range of 20° to 80°. The ultraviolet-visible (UV-vis) absorption spectra of the NCs were measured using a Jasco V-770 double-beam spectrophotometer, covering a wavelength range of 190 to 2700 nm. The morphology of NCs was checked by transmission electron microscopy (TEM, Joel-JEM 1010) operating at 80 kV. Photoluminescence (PL) spectra were acquired using the spectrophotometric system FLS1000 with a 450 W Xe lamp and the MicroSpec-2300i spectrometer with a He–Cd laser as the excitation source, and the excitation power (*P*_ex_) was adjusted from 5 × 10^−4^ to 5.6 mW. The Raman scattering (RS) spectra of the samples were recorded using a LABRAM-HR800 spectrometer (Jobin Yvon) with a wavelength of *λ* = 488 nm. X-ray photoelectron spectroscopy (XPS) was performed using a Thermo VG Escalab 250 photoelectron spectrometer.

## Results and discussion

### Morphological

**Fig. 2:**
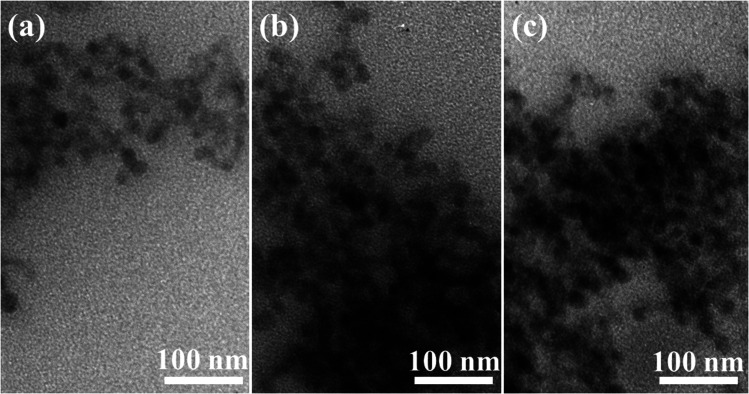
TEM images of NCs: (a) CoAl_2_O_4_, (b) Er1%-doped CoAl_2_O_4_, and (c) Er5%-doped CoAl_2_O_4_.

TEM images of three typical CoAl_2_O_4_, Er1%-doped CoAl_2_O_4_, and Er5%-doped CoAl_2_O_4_ samples are shown in Fig. 2. TEM images showed that the obtained NCs were a nearly spherical shape with fairly uniform sizes. The sizes of the particles were mostly in the range of 30 to 40 nm. The observation results show that Er doping did not significantly change the size and shape of the Er-doped CoAl_2_O_4_ alloy NCs.

### X-ray diffraction studies

**Fig. 3:**
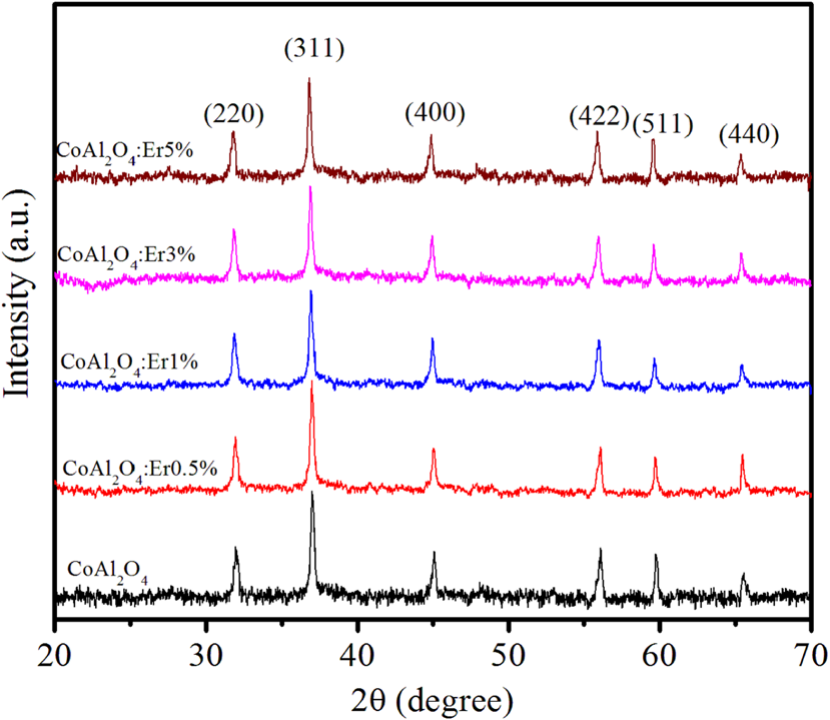
XRD patterns of Er-doped CoAl_2_O_4_ nanocrystals with varying Er concentrations.

**Fig. 4:**
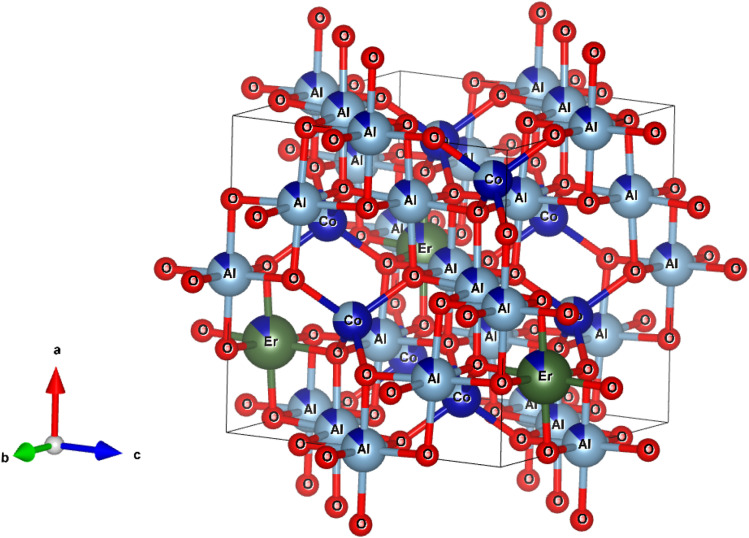
The unit-cells scheme of Er-doped CoAl_2_O_4_ with spinel structure.

Fig. 3 shows the XRD patterns of the CoAl_2_O_4_ and Er-doped CoAl_2_O_4_ NCs with different Er concentrations. All the nanocrystals (NCs) exhibited single-phase crystallization with a spinel structure (card JCPDS No. 44-0160) and a space group of *Fd*3̄*mz*.^18^ In the case of pure CoAl_2_O_4_ NCs, the diffraction peaks observed at 2*θ* angles correspond to 31.89, 36.68, 45.06, 56.27, 59.86, and 65.84°, indicating the lattice planes (220), (311), (400), (422), (511) and (440) of CoAl_2_O_4_. After doping with Er, only a CoAl_2_O_4_ spinel structure was obtained, with no detectable presence of Er oxide. This suggests that all Er was successfully incorporated into the CoAl_2_O_4_ lattice, without generating any oxide. The diffraction peaks of the Er-doped CoAl_2_O_4_ NCs shifted slightly toward smaller 2*θ* angles with increasing Er concentration. This result can be explained by the replacement of Al^3+^ with Er^3+^ ions to produce CoAl_2−*x*_Er_*x*_O_4_ alloy NCs. Er^3+^ ions replaced Al^3+^ ions in the formation of the CoAl_2−*x*_Er_*x*_O_4_ alloy structure (see in Fig. 4) because of the principle of preferential substitution based on similar valence states and analysis of element valence states.^4,19^

According to Pauling's rule, Er has a coordination number of 6, resulting in the formation of an octahedral structure denoted as ErO_6_. The radii of ions Co^2+^, Al^3+^, and Er^3+^ are 0.54, 0.675, and 1.03 Å, respectively.^19^ Owing to the significantly larger ionic radius of Er^3+^ compared to Co^2+^, an increase in the Er doping concentration results in an expansion of the lattice parameters of CoAl_2_O_4_. This observation strongly suggests that Er doping led to lattice expansion. The effective crystallite strain in the NCs was determined using the Stokes–Wilson equation:^20^10
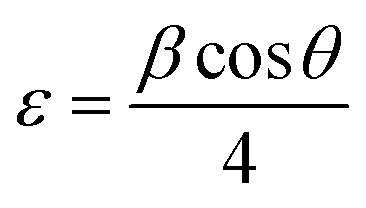
where *θ* is the diffraction angle, and *β* is the broadening of the diffraction line (measured at half of its maximum intensity). The average crystallite size (*D*) of the NCs was calculated using Debye–Scherrer equation:^21^11
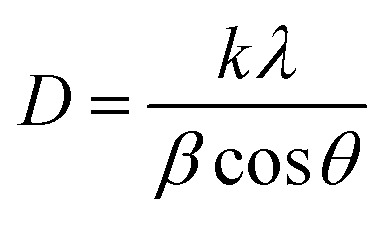
where *k* is the constant and usually a value of ∼0.9, *λ* is the wavelength of the Cu = K_α_ radiation (∼1.54 Å), The average crystallite size of the NCs was calculated based on the line broadening of the (311) peak.

The unit cell parameters of the Er-doped CoAl_2_O_4_ NCs at different Er concentrations were calculated using the following equation:^20,21^12
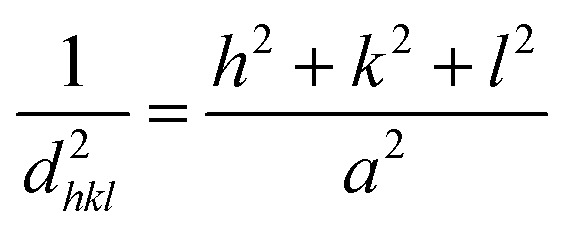
where *h*, *k* and *l* are the Miller indices, *d*_*hkl*_ and *a* are the interplanar spacing of the crystal and the lattice parameter, respectively. *d*_*hkl*_ was calculated using Bragg's equation:^20,21^

13 *nλ* = 2*d*_*hkl*_ sin *θ*

The unit cell volume of the NCs with a cubic structure was calculated using the following equation:

14 *V = a*^3^

The crystal lattice parameters of the Er-doped CoAl_2_O_4_ NCs NCs are calculated and given in Table 1.

**Table 1:** The diffraction angle (2*θ*), lattice constants (*a*), cell volume (*V*), *β*, crystallite size (*D*), crystallite strain (*ε*) of NCs

Sample	*2θ* (311)	*β* × 10^−2^ (rad)	*a* (Å)	*V* (Å^3^)	*D* (nm)	ε × 10^−3^
CoAl_2_O_4_	36.831	0.575	8.094	530.360	25.421	1.364
CoAl_2_O_4_:Er^3+^0.5%	36.712	0.584	8.095	530.442	25.034	1.386
CoAl_2_O_4_:Er^3+^1%	36.654	0.596	8.097	530.810	24.505	1.414
CoAl_2_O_4_:Er^3+^3%	36.530	0.583	8.100	531.344	25.065	1.384
CoAl_2_O_4_:Er^3+^5%	36.473	0.579	8.107	532.823	25.235	1.375

### Elemental and chemical composition analysis

**Fig. 5:**
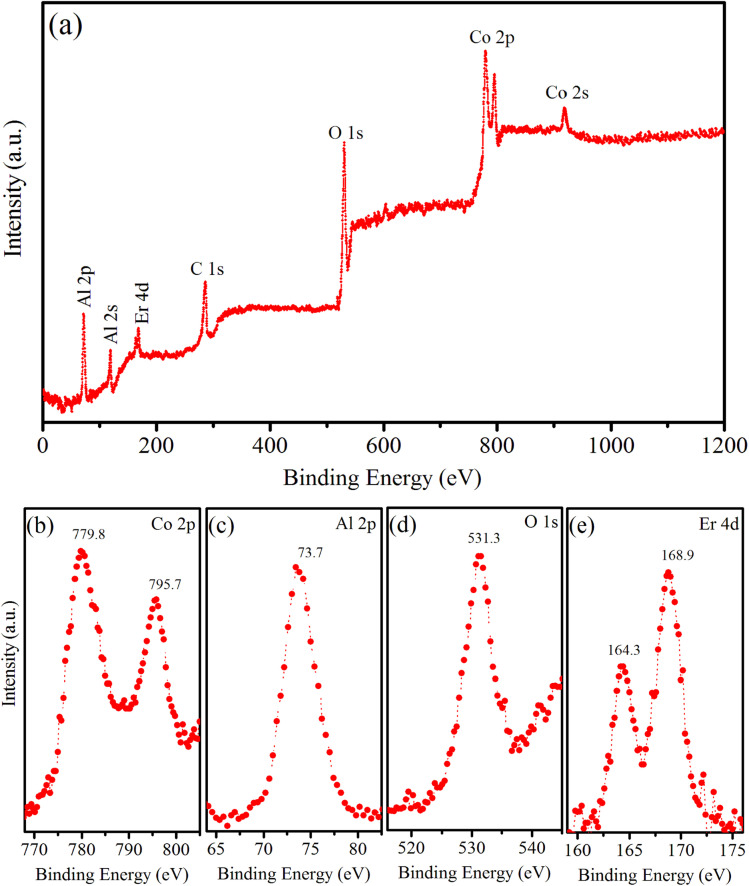
(a) Survey XPS spectrum of Er5%-doped CoAl_2_O_4_ NCs, (b) Co 2p, (c) Al 2p, (d) O 1s, and (e) Er 4d.

XPS is a versatile analytical technique that provides valuable information regarding the elemental composition, chemical state, electronic structure, and depth profiles of materials. In our study, XPS was used to analyze the chemical states of the elements present in the surface region of the synthesized sample, namely the Er5%-doped CoAl_2_O_4_ NCs. The results are shown in Fig. 5. Fig. 5a shows typical XPS survey scans of the Er1%-doped CoAl_2_O_4_ sample. The XPS survey spectrum suggests that, apart from the original components, only traces of contaminated carbon were detected, with no evidence of any other elements. The XPS survey scan shows seven peaks corresponding to the levels of Al-2p, Al-2s, Er-4d, C-1s, O-1s, Co-2p, and Co-2s. Fig. 5b shows the high-resolution XPS spectrum of Co-2p. It has two peaks centered at 779.8 and 795.7 eV, which correspond to Co-2p_3/2_ and Co-2p_1/2_, respectively. The energy difference between the two peaks is 15.9 eV, which is a characteristic feature indicative of the presence of Co^2+^ ions. The Co-2p spectrum is relatively narrow and symmetrical, indicating that Co^2+^ occupies octahedral sites in the synthesized samples.^22^Fig. 5c shows the high-resolution XPS spectrum of Al-2p. According to the study by Duan *et al.*,^23^ the Al-2p binding energies for octahedral and tetrahedral Al^3+^ ions are reported as 74.13 and 73.26 eV, respectively. In our study, the Al-2p binding energy peak fell within this range, suggesting the distribution of Al^3+^ ions among the two distinct sites within the CoAl_2_O_4_ NCs. The binding energy peak at 531.3 eV (Fig. 5d) is indexed to the O-1s level. The O-1s peak exhibits asymmetry and the binding energy of O-1s is similar to that of O in bulk CoAl_2_O_4_, as reported by Patterson *et al.*^24^Fig. 5e shows a high-resolution Er 4d XPS spectrum. The presence of two peaks at 164.3 and 168.9 eV indicates the Erbium in the trivalent state within the Er-doped CoAl_2_O_4_ NCs.^4,19^

### Absorption spectra and Judd–Ofelt analysis

**Fig. 6:**
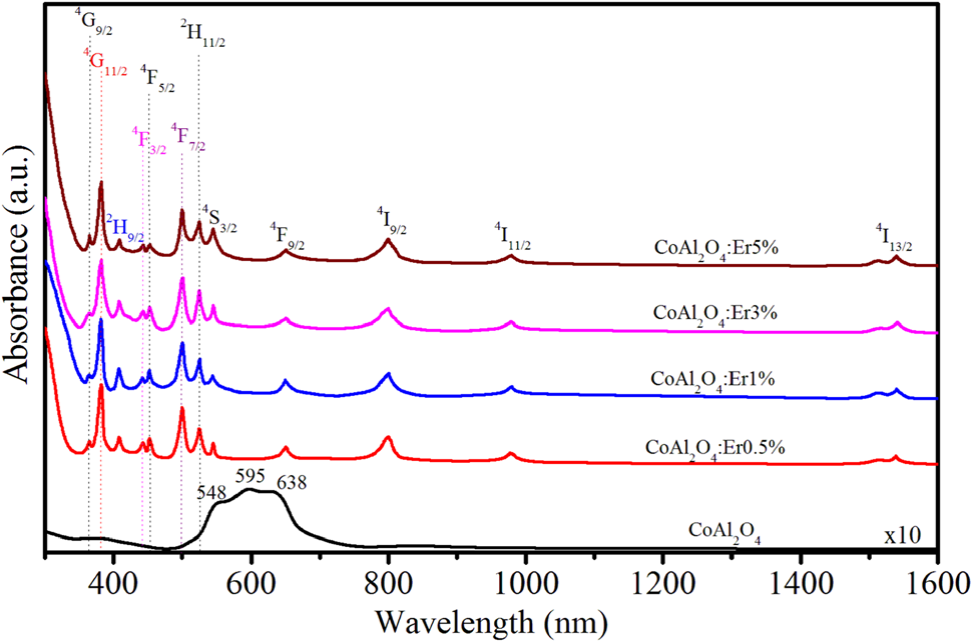
Absorption spectra of Er-doped CoAl_2_O_4_ NCs with varying Er concentrations.

To investigate the relationship between the cation distribution and optical characteristics of the NCs, we conducted measurements of the absorption spectra of the samples. The optical absorption spectra of the CoAl_2_O_4_ and Er-doped CoAl_2_O_4_ NCs were measured in the 300–1600 nm range, as shown in Fig. 6. The absorption spectra of the CoAl_2_O_4_ NCs showed three characteristic absorption peaks at 548, 595, and 638 nm, which indicate of the presence of Co^2+^ ions arranged in a ligand field with a 3d^7^ electron configuration.^25^ According to the literature, these three peaks are attributed to ^4^A_2_(F) → ^4^T_1_(P) transitions that arise due to the Jahn–Teller distortion of the Td structure, as indicated by ref. 26. The absorption spectra of the Er-doped CoAl_2_O_4_ NCs showed 12 peaks, corresponding to the transitions from the ground level to excited levels of Er^3+^ ion:^14–16^^4^I_15/2_–^4^G_9/2_, ^4^I_15/2_–^4^G_11/2_, ^4^I_15/2_–^2^H_9/2_, ^4^I_15/2_–^4^F_3/2_, ^4^I_15/2_–^4^F_5/2_, ^4^I_15/2_–^4^F_7/2_, ^4^I_15/2_–^2^H_11/2_, ^4^I_15/2_–^4^S_3/2_, ^4^I_15/2_–^4^F_9/2_, ^4^I_15/2_–^4^I_9/2_, ^4^I_15/2_–^4^I_11/2_, and ^4^I_15/2_–^4^I_13/2_. These peaks were centered at 365, 383, 413, 441, 452, 497, 520, 543, 651, 797, 978, and 1540 nm, respectively.

The formula for estimating the intensity of a transition in RE^3+^ ions based on the oscillator strength from the absorption spectra is as follows:^27,28^15
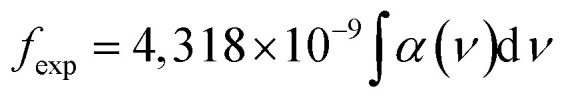
where *α*(*ν*) is molar extinction coefficient at energy *ν* (cm^−1^). The values of *α*(*ν*) can be calculated from absorbance *A* by using Lambert–Beer's law:^27,28^

16 *A* = *α*(*ν*)*cd*

where *c* is concentration (mol dm^−3^), *d* is the optical path length (cm).

In the case of RE^3+^ ions, Judd–Ofelt (J–O) theory has proven to be a valuable tool for estimating both the nature of the ligand field and their radiative properties. The cornerstone of J–O theory is a set of three intensity parameters, denoted as *Ω*_*λ*_ (*λ* = 2, 4, 6), which are related to the oscillator strength through the following expression:^27,28^17
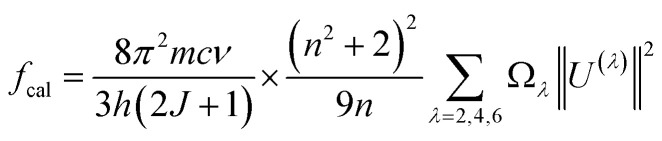
where *n* represents the refractive index of the material, *J* stands for the total angular momentum of the ground state, *Ω*_*λ*_ (*λ* = 2, 4, 6) are the intensity parameters, and ‖*U*_*λ*_‖^2^ are the squared doubly reduced matrix elements of the unit tensor operator with a rank of *λ* = 2, 4, 6. These matrix elements are essentially host matrix-independent and can be found in ^27^. The refractive index of the CoAl_2_O_4_ host (*n*) is approximately 1.4 and is considered constant across all wavelengths.^29^ The intensity parameters for the Er^3+^ ions in the CoAl_2_O_4_ NCs were determined using the least squares method by solving the equation *f*_cal_ = *f*_exp_. The obtained results have been presented in Table 2 for comparison with the values associated with other matrices.

**Table 2:** *f*_exp_, *f*_cal_ (×10^−6^) for excited levels of Er^+3^ ions in the Er-doped CoAl_2_O_4_ NCs

Transition from ^4^I_15/2_ to	*λ* (nm)	CoAl_2_O_4_:Er^3+^0.5%		CoAl_2_O_4_:Er^3+^1%		CoAl_2_O_4_:Er^3+^3%		CoAl_2_O_4_:Er^3+^5%	
		*f*_exp_	*f*_cal_	*f*_exp_	*f*_cal_	*f*_exp_	*f*_cal_	*f*_exp_	*f*_cal_
^4^G_9/2_	365	1.56	1.43	1.21	1.14	0.89	1.05	0.94	1.13
^4^G_11/2_	383	10.92	10.9	10.67	10.74	10.65	10.11	10.35	10.42
^2^H_9/2_	413	4.45	4.36	4.47	4.61	4.52	4.61	4.21	4.38
^4^F_3/2_	441	0.1	0.23	0.13	0.2	0.32	0.49	0.43	0.31
^4^F_5/2_	452	0.36	0.52	0.27	0.35	0.31	0.56	0.62	0.53
^4^F_7/2_	497	1.21	1.46	1.3	1.53	1.17	1.23	1.47	1.32
^2^H_11/2_	520	5.67	5.54	5.83	5.74	5.91	5.62	5.89	6.83
^4^S_3/2_	543	0.19	0.32	0.22	0.43	0.26	0.47	0.37	0.33
^4^F_9/2_	651	1.52	1.32	1.63	1.52	1.87	1.58	1.63	1.61
^4^I_9/2_	797	0.27	0.28	0.21	0.27	0.25	0.24	0.28	0.26
^4^I_11/2_	978	0.34	0.36	0.41	0.44	0.37	0.38	0.4	0.34
^4^I_13/2_	1540	0.96	0.63	1.24	1.02	1.27	1.22	1.08	1.12

Judd–Oflet intensity parameters (*Ω*_2_, *Ω*_4_, and *Ω*_6_) were determined from the absorption spectra using the least square fitting method. It is known that the *Ω*_2_, *Ω*_4_, and *Ω*_6_ parameters can provide valuable insights into the local coordination around RE^3+^ ions.^30–33^ The *Ω*_2_ parameter is related to the covalency of the bond between RE^3+^ and ligands, as well as ligand asymmetry, with larger *Ω*_2_ values indicating stronger binding. This is related to the electric-dipole transitions. Meanwhile, *Ω*_4_ and *Ω*_6_ are affected by factors such as viscosity and hardness but are almost unaffected by the local environment. Furthermore, *Ω*_6_ exhibits an inverse relationship with the covalent nature of the (Er–O) bonds. To evaluate the stimulated emission in the laser medium, a spectroscopic quality factor, denoted as *R* and defined as *R* = *Ω*_4_/*Ω*_6_, was introduced. *R* is important for predicting the potential performance of lasers.^31,32^ In Er^3+^-doped samples, *R* typically falls within the range of 0.33–2.94.^30–37^ This study revealed a decreasing trend in *R* with an increasing concentration of Er^3+^ ions. The J–O parameters (*Ω*_2_, *Ω*_4_, *Ω*_6_, and *R*) were calculated and compared with those of the other host materials as shown in Table 3. This study shows that the order of the Judd–Oflet parameters is *Ω*_2_ > *Ω*_6_ > *Ω*_4_ when the Er doping concentration changes. This trend is similar to that of other host materials doped with Er^3+^ ions, such as LaF_3_, phosphate, ErF_3_ (8% mol), and tellurite,^30–33^ but it is different from host materials doped with Er^3+^ ions, such as germanate, NaYF_4_, antimony, and LYB crystal.^34–37^ From the results shown in Table 3, we can see that the values of the *Ω*_*λ*_ parameters decrease with an increase in Er concentration. This suggests that the rigidity and local symmetry of the host materials become weaker as the concentration of the Er^3+^ ions increases. The highest value of the *Ω*_2_ parameter, when compared with *Ω*_4_ and *Ω*_6_, suggests that the vibrational frequencies in the given samples are relatively low.^31,32,34^

**Table 3:** Judd–Ofelt parameters: *Ω*_2_, *Ω*_4_, *Ω*_6_ (×10^−20^ cm^2^) and *R* of Er^+3^ in the samples

Sample	*Ω*_2_	*Ω*_4_	*Ω*_6_	*R*	Trend	Reference
CoAl_2_O_4_:Er^3+^0.5%	2.73	0.84	0.95	0.88	*Ω*_2_ >*Ω*_6_ > *Ω*_4_	Present work
CoAl_2_O_4_:Er^3+^1%	2.59	0.56	0.72	0.78	*Ω*_2_ >*Ω*_6_ > *Ω*_4_	Present work
CoAl_2_O_4_:Er^3+^3%	2.32	0.42	0.61	0.69	*Ω*_2_ >*Ω*_6_ > *Ω*_4_	Present work
CoAl_2_O_4_:Er^3+^5%	2.24	0.35	0.52	0.67	*Ω*_2_ >*Ω*_6_ > *Ω*_4_	Present work
LaF_3_	1.27	0.28	0.63	0.44	*Ω*_2_ >*Ω*_6_ > *Ω*_4_	30
Phosphate	3.91	1.97	2.57	0.76	*Ω*_2_ >*Ω*_6_ > *Ω*_4_	31
Tellurite	4.93	1.30	1.31	0.99	*Ω*_2_ >*Ω*_6_ > *Ω*_4_	32
ErF3 (8%mol)	0.61	0.14	0.43	0.33	*Ω*_2_ >*Ω*_6_ > *Ω*_4_	33
Germanate	4.81	1.41	0.48	2.94	*Ω*_2_ >*Ω*_4_ > *Ω*_6_	34
NaYF_4_	2.16	1.40	0.64	2.19	*Ω*_2_ >*Ω*_4_ > *Ω*_6_	35
Antimony	4.05	1.14	0.73	1.56	*Ω*_2_ >*Ω*_4_ > *Ω*_6_	36
LYB crystal	7.67	1.45	0.82	1.77	*Ω*_2_ >*Ω*_4_ > *Ω*_6_	37

### Photoluminescence spectra and CIE color coordinates

**Fig. 7:**
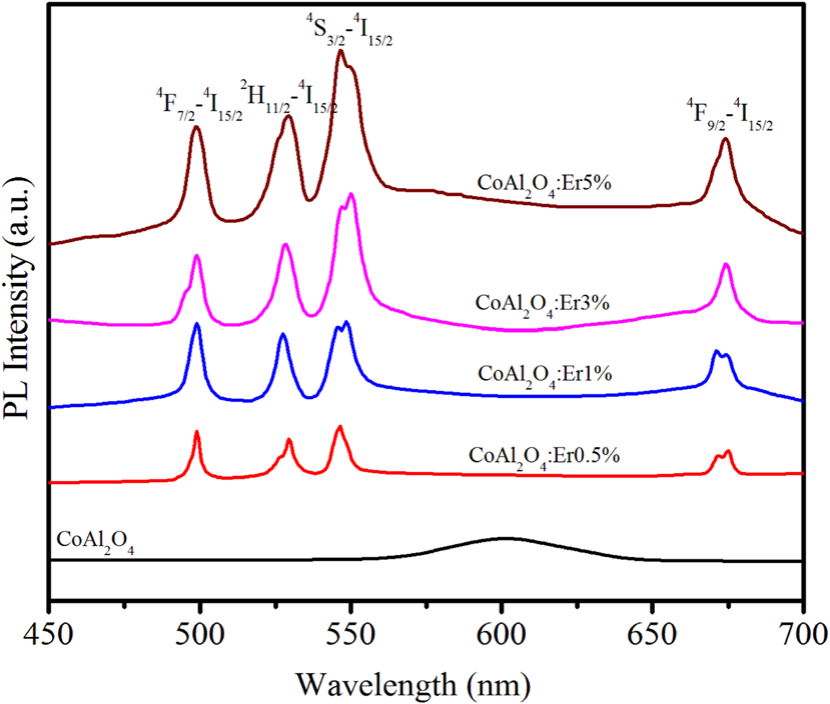
PL spectra of Er-doped CoAl_2_O_4_ nanocrystals with varying Er concentrations.

The PL spectra of the samples in the wavelength range of 450–700 nm with an excitation wavelength of 413 nm (^4^I_15/2_–^2^H_9/2_ transition) are observed in Fig. 7. For pure CoAl_2_O_4_ NCs, the emission spectrum exhibited a peak at 602 nm. This emission peak is attributed to the ^2^E(^2^G) → ^4^A_2_(^4^F) transition of tetrahedral Co^2+^ ions^38^ and provides clear evidence of the presence of tetrahedral Co^2+^ ions within the spinel structure of the CoAl_2_O_4_ NCs.^39^ The PL spectra of the Er-doped CoAl_2_O_4_ NCs exhibit four distinct emission peaks associated with the transitions of Er^3+^ ions at wavelengths of 502, 533, 548, and 678 nm. These transitions correspond to the shifts ^4^F_7/2_–^4^I_15/2_, ^2^H_11/2_–^4^I_15/2_, ^4^S_3/2_–^4^I_15/2_, and ^4^F_9/2_–^4^I_15/2_.^15,40^ As shown in Fig. 7, the intensity of these emission peaks increased proportionally with the concentration of Er^3+^. This observation confirms that no fluorescence quenching occurred in the Er-doped CoAl_2_O_4_ NCs, even when the Er ion concentration reached 5%.

**Fig. 8:**
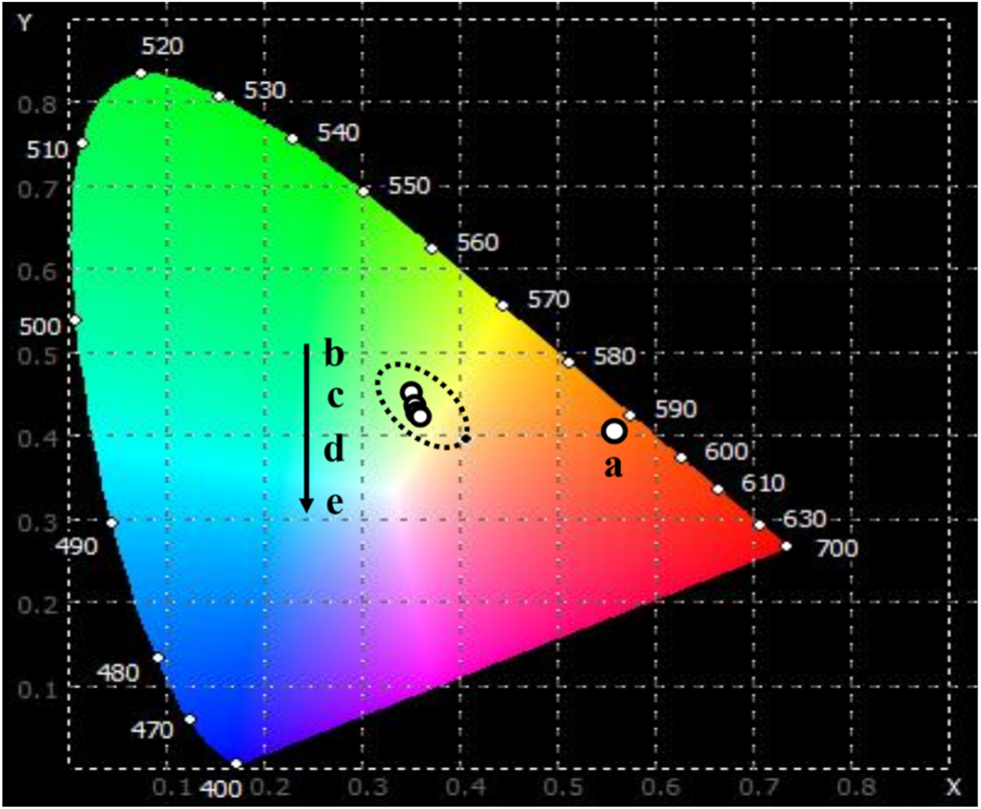
The CIE color coordinates diagram of CoAl_2_O_4_:*x*%Er^3+^ NCs: *x* = 0 (a), 0.5 (b), 1.0 (c), 3.0 (d), and 5.0 (e) with *λ*_exc_ = 413 nm.

The emission characteristics of a substance are commonly determined using the CIE chromaticity coordinates. Utilizing the emission spectra resulting from the excitation at 413 nm, the color coordinate diagram of the Er-doped CoAl_2_O_4_ NCs was estimated and is illustrated in Fig. 8. The corresponding (*x*, *y*) coordinates are listed in Table 4. In the case of the pure CoAl_2_O_4_ NCs, the luminescence spectrum encompasses emission bands within the red-orange region, as depicted in Fig. 8. For the Er-doped CoAl_2_O_4_ NCs, the emission color shifted strongly toward the blue and yellow regions. It can be seen that the emission color of the Er-doped CoAl_2_O_4_ NCs changes insignificantly when the Er concentration increases from 0.5–5%. The correlated color temperature (CCT) (K) of a material was determined using the following formula:^41^

18 CCT = −449*n*^3^ + 3525*n*^2^ − 6823*n* + 5520.33

where *n* is calculated using the expression: *n* = (*x* − *x*_e_)/(*y* − *y*_e_) with *x*_e_ = 0.332 and *y*_e_ = 0.186. The calculated CCT values for the samples are listed in Table 4. These CCT values correspond to neutral white light as perceived by the human visual system.^42^

**Table 4:** The chromaticity coordinates (*x*, *y*) and the correlated color temperature (CCT) for CoAl_2_O_4_:*x*%Er^3+^ (*x* = 0–5%) NCs

Sample	*x*	*y*	CCT
CoAl_2_O_4_	0.560	0.400	1709
CoAl_2_O_4_:0.5%Er^3+^	0.358	0.463	4911
CoAl_2_O_4_:1.0%Er^3+^	0.360	0.423	4763
CoAl_2_O_4_:3.0%Er^3+^	0.365	0.422	4634
CoAl_2_O_4_:5.0%Er^3+^	0.361	0.420	4728

### Upconversion fluorescence

**Fig. 9:**
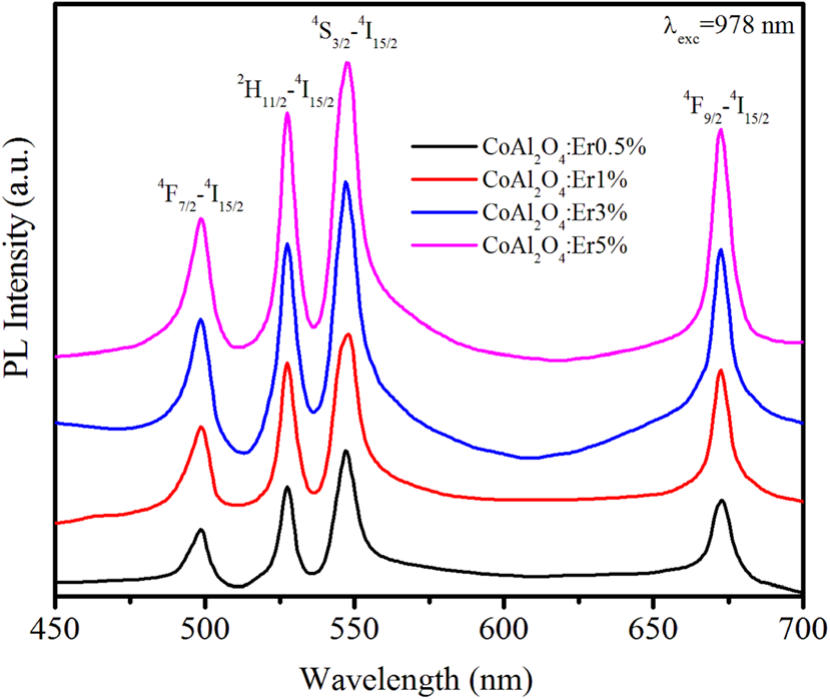
The up-conversion emission spectra of Er-doped CoAl_2_O_4_ nanocrystals with varying Er concentrations, recorded at *λ*_exc_ = 978 nm.

Up-conversion (UC) emission is a photophysical phenomenon in which a material absorbs two or more lower-energy photons and subsequently emits a single higher-energy photon. The UC luminescence phenomenon has garnered significant attention from numerous researchers due to its multiple promising applications in fields such as bio-imaging, display technology, solar cells, medical diagnostics, and photodynamic therapy. Fig. 9 shows the up-conversion PL spectra of the Er-doped CoAl_2_O_4_ NCs (obtained at *λ*_exc_ = 978 nm) in the spectral region of 450–700 nm.

The Er^3+^ ions in the ground state were excited and transitioned to the ^4^I_11/2_ state. The energy transfer process occurs between Er^3+^ ions according to the following equation:

19 ^4^I_11/2_ + ^4^I_11/2_ → ^4^I_15/2_ + ^4^F_7/2_

This energy transfer process causes the Er^3+^ ions to transition to the ^4^F_7/2_ excited state. The ions in the ^4^F_7/2_ state recover to the ground state and emit light from violet to the infrared region.^43^ The peak positions of all the observed emission bands were almost unchanged compared to the observations in Fig. 7 (*λ*_exc_ = 413 nm). The most intense emission peaks are observed at 533 nm (^2^H_11/2_–^4^I_15/2_) and 548 nm (^4^S_3/2_ → ^4^I_15/2_).

**Fig. 10:**
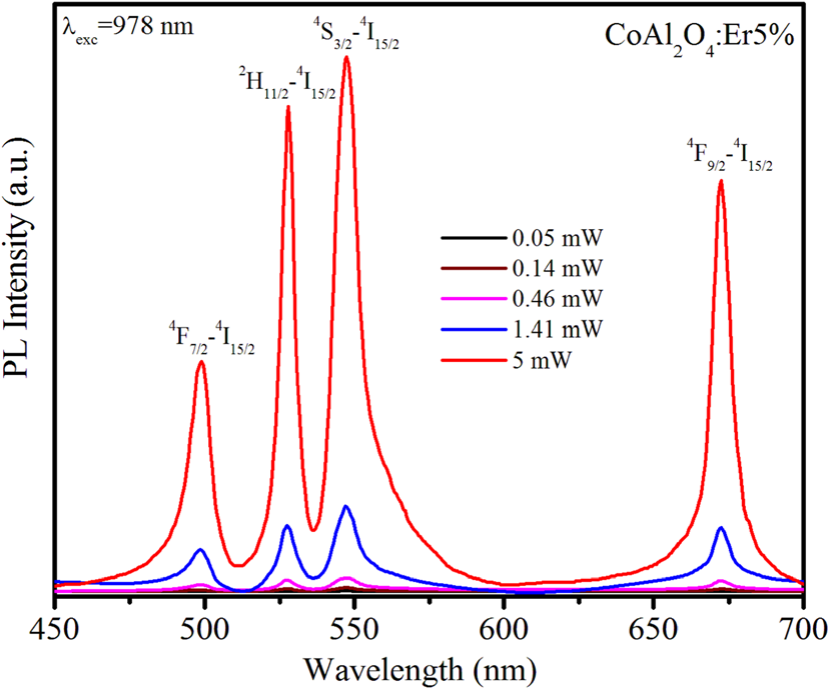
Upconversion emission spectra of Er5%-doped CoAl_2_O_4_ nanocrystals when excited at 978 nm (^4^I_15/2_–^4^I_11/2_) with varying excitation power from 0.05 to 5 mW.

The UC phenomenon has been explained by several physical mechanisms such as Auger recombination, two-photon absorption, two-step two-photon absorption, and the thermal excitation of surface states. To gain a deeper understanding of the UC mechanism, we investigated the power-dependent luminescence. Fig. 10 displays the UC emission spectra of Er5%-doped CoAl_2_O_4_ NCs when excited at 978 nm with varying excitation power (from 0.05 to 5 mW).

As the excitation power increased, the emission intensity of Er^3+^ increased. The relationship between the up-conversion luminescence intensity (*I*_uc_) and excitation power (*P*) can be determined using the following formula:^44^

**Fig. 11:**
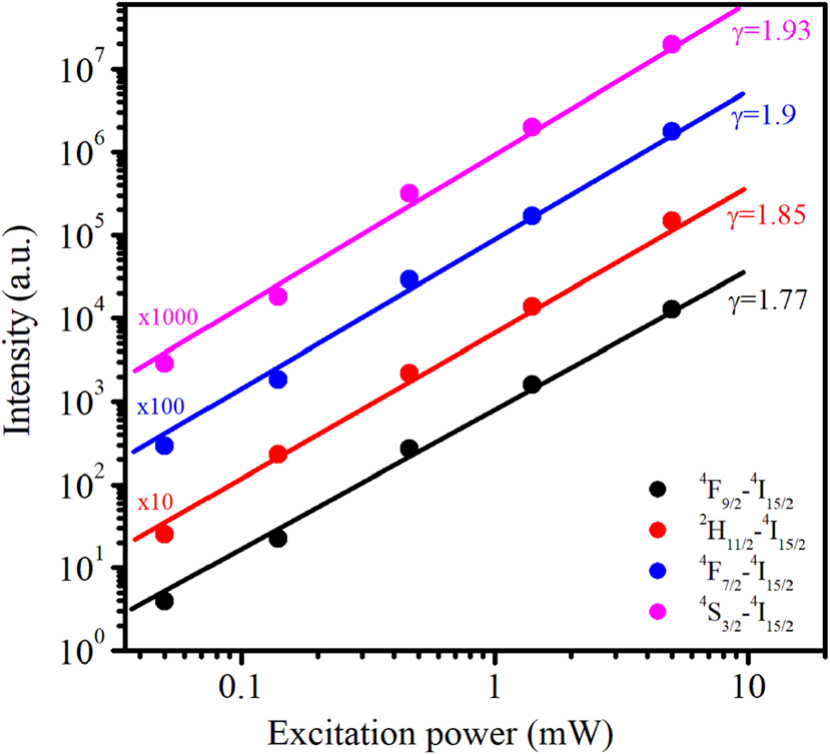
The dependence of upconversion emission intensity on excitation power (from 0.05 to 5 mW) under 978 nm excitation for Er1%-doped CoAl_2_O_4_ nanocrystals.

20 *I*_uc_ ∼ *P*^*n*^

where *I*_uc_ is the fluorescence intensity of the Er^3+^ ions, *P* is the excitation power, and *n* is the number of photons required to generate the excited state. The dependence of ln *I*_uc_ on ln*P* for these transitions is shown in Fig. 11. These experimental data can be well-fitted linearly, and the obtained *n* values are 1.9, 1.85, 1.93, and 1.77 for ^4^F_7/2_–^4^I_15/2_, ^2^H_11/2_–^4^I_15/2_, ^4^S_3/2_–^4^I_15/2_, and ^4^F_9/2_–^4^I_15/2_ transitions, respectively. These obtained *n* values are approximately equal to 2 indicating that the UC mechanism of these transitions is the result of a two-photon absorption process.^44–46^ The two-photon absorption mechanism was also used to explain the upconversion fluorescence phenomenon in Er^3+^ and Yb^3+^-codoped YBO_3_, and Er^3+^-doped YbOCl samples.^45^ The UC mechanism can be attributed to the saturation phenomenon in which the *n* values of the transitions were significantly smaller (0.86–1.58) than the theoretical values.^46^

**Fig. 12:**
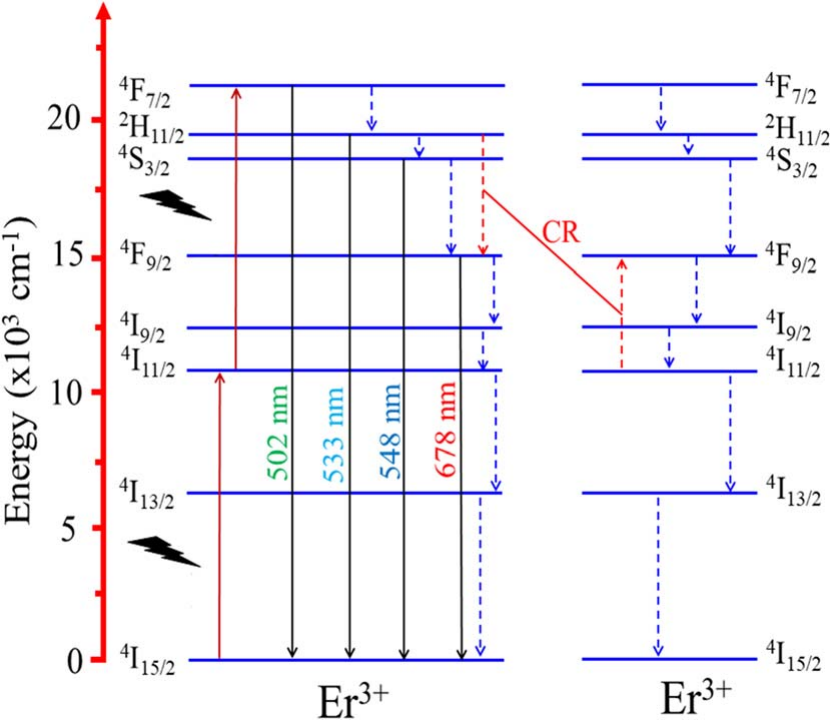
Energy level diagrams of Er^3+^, showing possible energy transfer processes in the Er-doped CoAl_2_O_4_ NCs.

The two-photon absorption mechanism of UC emission is explained by the diagram in Fig. 12. Initially, Er^3+^ ions absorb photons, transitioning from the ground level ^4^I_15/2_ to the ^4^I_11/2_ level. Subsequently, the absorption of the second photon raises it from ^4^I_11/2_ level to ^4^F_7/2_ level. From ^4^F_7/2_ state, it recovers to ^4^I_15/2_ state by emitting a photon with a wavelength of 502 nm and recovers the multiphonon to the ^2^H_11/2_, ^4^S_3/2_, and ^4^F_9/2_ states. These transitions produce emission peaks at 533, 548, and 678 nm corresponding to ^2^H_11/2_–^4^I_15/2_, ^4^S_3/2_–^4^I_15/2_, and ^4^F_9/2_–^4^I_15/2_ transitions (located in the blue and red light regions). It's worth noting that the ^4^S_3/2_ excited level possesses a longer intrinsic lifetime than the ^2^H_11/2_ level, increasing the probability of non-radiative transitions. Therefore, the observed results suggest that relaxation from the ^4^F_7/2_ state to the ^4^S_3/2_ state is the most favored transition.^47^

### Decay time curves of the ^4^S_3/2_–^4^I_15/2_ transition (548 nm)

**Fig. 13:**
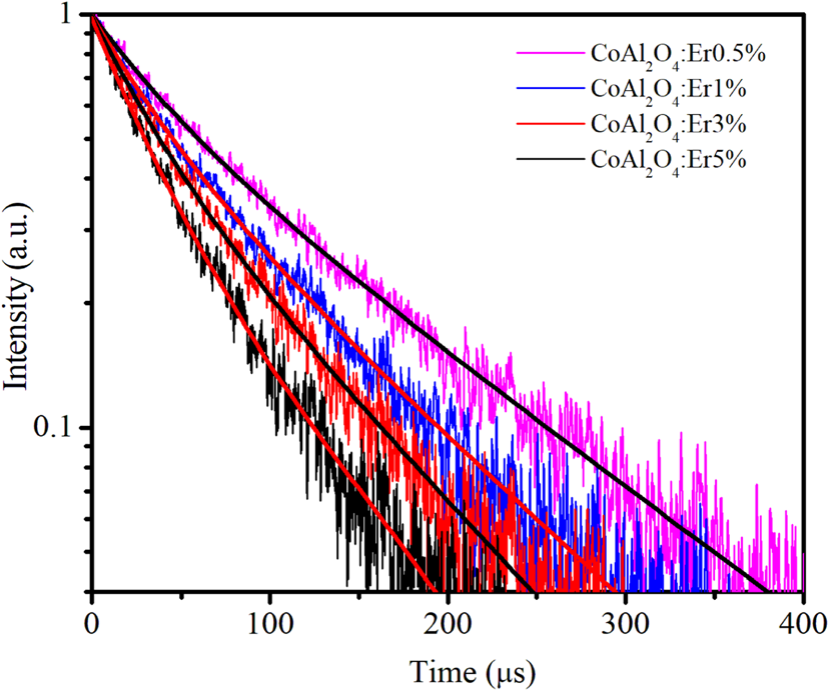
Decay time curves of Er-doped CoAl_2_O_4_ NCs measured at 548 nm (^4^S_3/2_–^4^I_15/2_), using an EPL-405 in the FLS1000. Measurement parameters: repetition rate = 200 kHz, excitation pulse width = 1 μs, *λ*_ex_ = 413 nm, Δ*λ*_ex_ = 5 nm, resolution = 10 ns per channel.

Time-resolved PL measurements provide insight into the decay times of the luminescence process. Fig. 13 shows the decay curves of the ^4^S_3/2_–^4^I_15/2_ transition (548 nm) for the four samples doped with Er^3+^, *λ*_ex_ = 413 nm. The emission of Er^3+^ ions in host materials can be attributed to various recombination mechanisms or energy transfer processes. The lifetimes of the ^4^S_3/2_–^4^I_15/2_ transition of the samples were determined by analyzing the PL decay curve, which was fitted using a bi-exponential function:^48^21
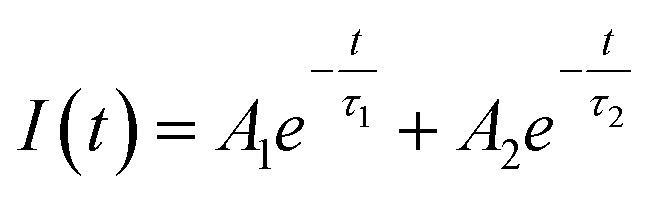
where *A*_*i*_ represents the magnitude and *τ*_*i*_ denotes the lifetime of the *i*_*th*_ component, which is known to be highly influenced by the structure, elemental composition, size, and morphology of the NCs.^49^ The average lifetime 〈*τ*〉 is determined based on *A*_*i*_ and *τ*_*i*_ according to the following equation:^50^22
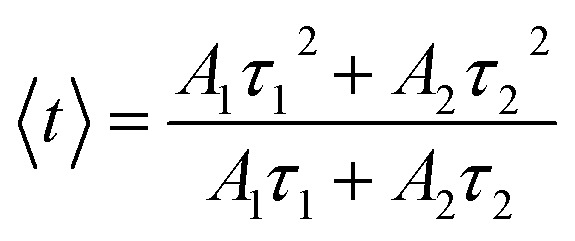


The fitting time constants for the ^4^S_3/2_–^4^I_15/2_ transition are presented in Table 5.

**Table 5:** Lifetime decay constants and fitness of curves

Sample	*τ*_1_ (μs)	*A*_1_	*τ*_2_ (μs)	*A*_2_	〈*τ*〉 (μs)	*R* ^2^
0.5%	39.69	0.342	135.45	0.658	122.79	0.9954
1%	35.53	0.416	109.83	0.584	95.92	0.9949
3%	32.53	0.465	95.56	0.535	81.17	0.9948
5%	32.48	0.673	105.7	0.327	77.33	0.9944

A continuous decrease in the average decay lifetime of the ^4^S_3/2_–^4^I_15/2_ transition at 548 nm was observed as the concentration of Er^3+^ increased. This finding suggests that the additional decay channels, which accelerate the reduction in the lifetime of the ^4^S_3/2_ excited state, become more influential at higher concentrations of Er^3+^. This phenomenon may be attributed to the heightened impact of nonradiative processes, energy migration among neighboring Er^3+^ ions, the presence of quenching centers, such as OH- groups in the glass host matrix, and cross-relaxation (CR), as illustrated in Fig. 12 (the red dashed line). The CR process plays a crucial role in efficiently compensating for the loss of excited energy at the ^4^F_9/2_ level through nonradiative relaxation, particularly at relatively large concentrations of Er^3+^.

## Conclusion

Er-doped CoAl_2_O_4_ NCs with sizes of approximately 30–40 nm were successfully synthesized using the co-precipitation method. The effect of Er doping concentration on the color, structure, and optical properties of the CoAl_2_O_4_ NCs was studied. The XRD pattern shows that the CoAl_2_O_4_ and Er-doped CoAl_2_O_4_ NCs have a spinel structure and do not exhibit any secondary phases. The absorption spectra of the Er-doped CoAl_2_O_4_ NCs showed 12 characteristic absorption peaks of Er ions at 365, 383, 413, 441, 452, 497, 520, 543, 651, 797, 978, and 1540 nm, respectively. The *Ω*_*λ*_ parameters were calculated and their values decreased with an increase in Er concentration. This suggests that the rigidity and local symmetry of the CoAl_2_O_4_ host materials became weaker as the concentration of Er^3+^ ions increased. The highest value of the *Ω*_2_ parameter suggests that the vibrational frequencies of the given samples are relatively low. The PL spectra of the Er-doped CoAl_2_O_4_ NCs showed four characteristic emission peaks at 502, 533, 548, and 678 nm originating from the f–f transition in Er^3+^ ions. Up-conversion PL spectra for Er-doped CoAl_2_O_4_ NCs were obtained using an excitation wavelength of 978 nm, with excitation powers ranging from 0.05 to 5 mW. The dependence of *I*_uc_ on *P*^*n*^ with *n* is approximately 2 indicating that the UC mechanism is the result of a two-photon absorption process. The decay time of the ^4^S_3/2_–^4^I_15/2_ transition decreased as the Er^3+^ concentration increased. The CR process plays an important role in reducing the energy loss at the ^4^F_9/2_ level through non-radiative relaxation, especially at high Er^3+^ concentrations. The research results obtained show that these Er-doped CoAl_2_O_4_ NCs are promising candidates for photonic and color printing applications. The creation of these novel ceramic pigments carries both scientific importance and practical utility.

## Conflicts of interest

There are no conflicts to declare.

## Supplementary Material
